# Prenatal and Early-Life Exposure to Microbiome-Modulating Medications and the Risk of Childhood Food Allergy: A Systematic Review and Meta-Analysis

**DOI:** 10.3390/jcm15083086

**Published:** 2026-04-17

**Authors:** Diána Bodó, Bettina Vargáné Szabó, Tivadar Kiss, Dezső Csupor, Barbara Tóth

**Affiliations:** 1 Institute of Clinical Pharmacy, Faculty of Pharmacy, University of Szeged, 6725 Szeged, Hungary; bodo.diana@szte.hu (D.B.); vargane.szabo.bettina@szte.hu (B.V.S.); 2 Institute of Pharmacognosy, Faculty of Pharmacy, University of Szeged, 6720 Szeged, Hungary; kiss.tivadar@szte.hu; 3 Institute of Translational Medicine, University of Pécs, 7624 Pécs, Hungary

**Keywords:** anti-bacterial agents, antacids, early-life exposure, food hypersensitivity, meta-analysis, prenatal exposure, probiotics, proton pump inhibitors

## Abstract

**Background/Objectives**: Several recent human studies have associated the use of certain medicines, such as antibiotics and antacids, with allergic conditions, potentially through microbiome disruption. In contrast, probiotics which may prevent dysbiosis, could have protective effects. Our meta-analysis aimed to evaluate the impact of these drugs (consumed during pregnancy or early life) on the risk of childhood food allergy, based on the available literature. **Methods**: Literature searches were conducted in the EMBASE, PubMed, Cochrane, and Web of Science databases using predefined PICO criteria. Overall, our meta-analysis included 25 studies involving 1,662,861 mothers and 5,164,280 children. **Results**: Using the random-effects model, we found that prenatal and early life antibiotic use (up to 2 years of age) was associated with higher odds of food allergy in childhood (OR: 1.34; 95% CI [1.10, 1.63], OR: 1.53; 95% CI [1.18, 1.98], respectively). Proton pump inhibitors were also associated with a risk of food allergies (OR: 2.65; 95% CI [1.22–5.77]), whereas the impact of H2-receptor antagonists was non-significant (OR: 2.07; 95% CI [0.96–4.45]). Probiotic use during the first two years of life was not associated with decreased risk for food allergy in children (OR: 1.25; 95% CI [0.46, 3.38]). **Conclusions**: These findings suggest an association between microbiome-disrupting medications during pregnancy and early childhood and an increased risk of childhood food allergy, especially those with a family history of food allergy. However, due to the predominantly observational design of the included studies, causality cannot be established. These results highlight the need for cautious and judicious use of such medications in these populations.

## 1. Introduction

The prevalence of food allergy in children has increased substantially over recent decades, representing a growing public health challenge [1,2,3]. Early immune development is critically shaped by the establishment of the gut microbiome, which plays a central role in the induction of oral tolerance to dietary antigens. Disruptions in the composition and diversity of the infant microbiota have been implicated in the pathogenesis of allergic diseases, including food allergy [1,2,3]. A number of commonly used medications, such as antibiotics, acid-suppressive medications and probiotics, are known to alter the microbiome and influence immune maturation [1,2,3].

Antibiotic exposure during pregnancy or early life has been associated with an increased risk and earlier onset of food allergy. Antibiotic-induced dysbiosis impairs normal microbial colonization and immune tolerance, thereby predisposing children to allergic diseases [4,5].

Acid-suppressive medications, including proton pump inhibitors (PPIs) and histamine-_2_-receptor antagonists (H_2_RAs) are also frequently used during pregnancy and early life [6]. By reducing gastric acidity, these medications impair protein digestion, alter the microbial composition, and increase the exposure of the immune system to intact dietary antigens. Several observational studies have reported associations between prenatal or postnatal acid-suppressive medication use and increased risk of food allergy [6].

Probiotic supplementation has also been widely investigated as a means of modulating the prenatal and early life microbiome [7]. The effects of probiotics on the incidence of food allergies have yet to be clarified. While several randomized trials and long-term follow-up studies have demonstrated no effect on food allergy incidence among children exposed to probiotics, some have reported increased rates of allergic sensitization in probiotic-exposed groups [7]. This highlights the complexity of microbiome manipulation during pregnancy and early life [7]. Taken together, antibiotics, acid-suppressive medications, and probiotics are considered common prenatal or postnatal exposures that may modify the infant microbiome. However, the impact of these microbiome-influencing medications on the risk of childhood food allergies remains to be determined. Although several systematic reviews and meta-analyses have examined the relationship between microbiome-modifying exposures and allergic outcomes, these studies have typically evaluated composite atopic conditions rather than focusing specifically on food allergy. Furthermore, to date, no meta-analysis has simultaneously assessed the effects of antibiotics, acid-suppressive medications and probiotics within a unified framework. In addition, existing studies rarely distinguish the timing of exposure with limited integration of prenatal, intrapartum and postnatal periods in a single comprehensive analysis. Moreover, few studies have explored potential differences across specific drug classes or subclasses, or evaluated the impact of exposure according to gestational timing, such as by trimester [8,9,10,11,12]. Therefore, the aim of the present study is to investigate the association between prenatal and early life use of microbiome-affecting medications and the development of food allergy in offspring [13,14].

## 2. Materials and Methods

### 2.1. Protocol

This meta-analysis was planned and performed following the PRISMA 2020 (Preferred Reporting Items for Systematic Reviews and Meta-Analyses) recommendations, and it was registered in the International Prospective Register of Systematic Reviews (PROSPERO) (registration number: CRD420261284798).

The following PICOS (patient, intervention, control, outcome, study design) framework was applied as follows: P: pregnant women or children during early life (up to 2 years of age), I: usage of microbiome-influencing medications including antibiotics, acid-suppressive medications ([PPIs] and [H_2_RAs]) and probiotics, C: individuals not exposed to these medications, O: development of food allergy in offspring until 10 years of age.

### 2.2. Information Sources and Search Strategy

A comprehensive literature search was conducted using the Embase, PubMed, Cochrane Central Register of Controlled Trials (CENTRAL) and Web of Science databases until 20 December 2025 using the following search strategy: ((“Anti-Bacterial Agents”[Mesh] OR antibiotic* OR antimicrobial*) OR (“Probiotics”[Mesh] OR probiotic*) OR (“Microbiota”[Mesh] OR microbiome OR microbiota OR dysbiosis) OR (“Antacids”[Mesh] OR “Proton Pump Inhibitors”[Mesh] OR “Histamine H2 Antagonists”[Mesh] OR antacid* OR “acid suppressant*” OR PPI OR “H2 receptor antagonist*”)) AND (“Food Hypersensitivity”[Mesh] OR “food allerg*” OR “food sensitiz*” OR atopy) AND ((“Pregnancy”[Mesh] OR prenatal OR maternal OR pregnancy OR gestation OR antenatal OR perinatal) OR (“Infant”[Mesh] OR “Child”, Preschool”[Mesh] OR “Infant, Newborn”[Mesh] OR infant* OR neonat* OR newborn* OR “early life”)).

No restrictions were applied with regard to language, publication date or publication status. In addition, the reference lists of all eligible studies were manually screened.

### 2.3. Eligibility Criteria and Study Selection

All studies that recorded exposure to microbiome-influencing medications during pregnancy or in children during early life (up to 2 years of age), either from databases or via questionnaires, and reported food allergy outcomes in the offspring until 10 years old were included. Zotero 7.0 software was used for reference management. After removing duplicates, the remaining records were screened for eligibility based on titles and abstracts. The full texts of the identified records were independently assessed for eligibility by two reviewers (D.B., B.T.). Any disagreements were resolved through discussion and if necessary, a third reviewer (D.C.) who had not been involved in the initial selection was consulted. Studies that did not meet the PICOS’s criteria, lacked numerical data on exposures or outcomes, or were not fully accessible in any form were excluded. The authors were not contacted for additional information. Although diagnostic approaches for food allergy varied across studies, all studies reporting food allergy as an outcome were included to maximize sample size and enhance the robustness of the analysis.

### 2.4. Data Extraction and Synthesis of the Results

Data extraction was performed according to PRISMA guidelines (Supplementary File S2). From the included studies, the following data were extracted: study design, characteristics of the patient population (pregnant women or children during early life [up to 2 years of age]), type of drug (e.g., antibiotics, acid-suppressive medications and probiotics) and timing (prenatal and postnatal) of the medication exposure, age at the time of the diagnosis of food allergy, source of exposure data (databases or questionnaires), mode of delivery and geographic region where the study was conducted. Food allergy outcomes were identified based on the definitions reported in the original studies, including diagnostic codes from administrative databases, allergen sensitization determined by serum specific immunoglobulin E, skin prick testing performed with commercial food allergens or physician-diagnosed food allergy reported in questionnaires. Food allergy outcomes were considered comparable across studies despite differences in diagnostic methods, as all definitions reflected clinically relevant or immunologically mediated food allergy outcomes.

### 2.5. Risk of Bias Analysis

The risk of bias of the included studies was assessed using appropriate tools according to study design. For observational studies evaluating antibiotic and acid-suppressive medication exposure, the Risk of Bias in Non-randomized Studies of Interventions (ROBINS-I) tool was applied [15]. Randomized controlled trials investigating probiotic use were assessed using the Cochrane Collaboration Risk of Bias tool. For each domain, studies were classified as having low, moderate/some concerns or high risk of bias. The results of the risk of bias assessments were visualized using traffic lights plots generated with the robvis tool, where green, yellow and red colors indicated low, moderate/some concerns and high risk of bias, respectively. The ROBINS-I tool was applied across the following domains: bias due to confounding, selection of participants, classification of interventions, deviations from intended interventions, missing data, measurement of outcomes and selection of reported result. Each domain was assessed independently, and overall study-level judgements were based on the highest risk identified across domains. Particular attention was given to confounding and exposure classification, which are especially relevant in observational studies of medication use.

### 2.6. Statistical Analysis

The statistical analysis was performed using RStudio meta and metafor packages (version 2024.9.0.375). A random-effects model was applied to account for variability between studies, aiming to estimate the mean of the distribution of effects. Pooled effect estimates were reported with 95% confidence intervals (CIs) and, where applicable, unadjusted odds ratios (ORs). When possible, effect estimates were recalculated from individual study data to ensure consistency, and no significant differences were observed compared to the reported values. Statistical heterogeneity was assessed using I^2^ an τ^2^, with I^2^ interpreted according to the Cochrane Handbook (low 0–40%, moderate 30–60%, substantial 50–90%, considerable 75–100%). Forest plots were generated for each study and pooled results, and data visualization was performed with metafor. Results from studies that could not be included quantitatively were synthesized qualitatively, narratively. We performed leave-one-out analyses for sensitivity analysis. To assess the heterogeneity between the studies, subgroup analysis was performed. Funnel plots and Egger’s test were used to evaluate potential publication bias and study asymmetry.

### 2.7. Quality of Evidence

The quality of evidence was assessed using the GRADE approach with GRADEpro GDT (Guideline Development Tool, accessed on February 2026). Each outcome was rated according to the following: risk of bias, inconsistency, indirectness, imprecision, publication bias, presence of a large effect, dose-dependent response and plausible confounders. The overall, certainty of evidence can be classified as very low, low, moderate, or high.

## 3. Results

### 3.1. Study Selection

**Figure 1:**
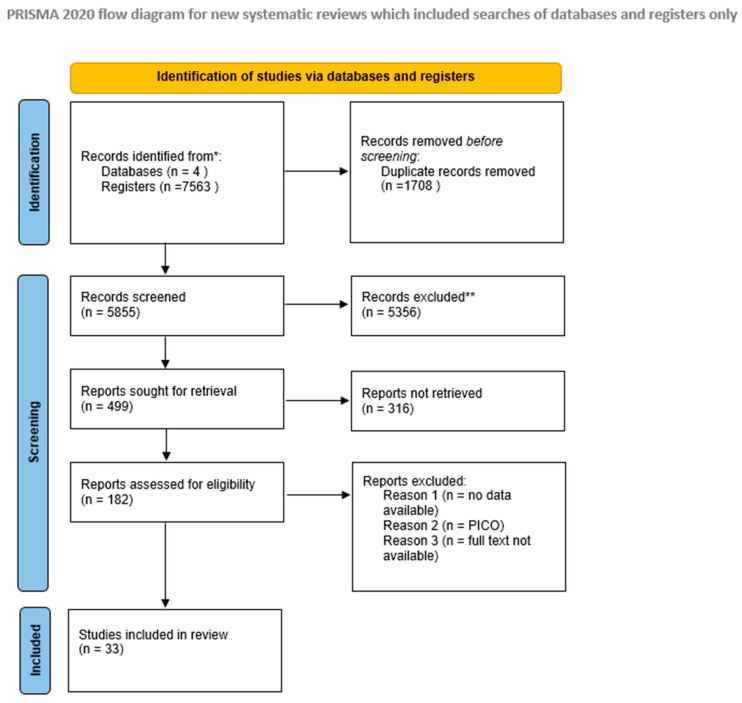
PRISMA flow chart. * Total number of records identified from EMBASE, Web of Science, PubMed, and CENTRAL. ** No automation tools were used in the selection process.

Initially, a total of 7563 records were identified through database searches. After removal of duplicates with Zotero, 5855 records remained for screening. Based on title screening, 5356 records were excluded, and the abstracts of the remaining 499 records were assessed (Cohen’s kappa = 0.83), resulting in the exclusion of 316 studies. Full-text screening was subsequently performed for 182 articles, of which 149 were excluded (Cohen’s kappa = 0.87). Ultimately, 33 studies met the inclusion criteria and were included in the meta-analysis. The study selection process is illustrated in the PRISMA flow diagram (Figure 1).

### 3.2. Study Characteristics

#### 3.2.1. Qualitative Analysis

A total of 33 studies [3,6,13,14,16,17,18,19,20,21,22,23,24,25,26,27,28,29,30,31,32,33,34,35,36,37,38,39,40,41,42,43,44] met the predefined PICOS criteria and were included in the meta-analysis. Of these, 27 studies were eligible for inclusion in the quantitative meta-analysis, while 8 studies [14,16,17,18,19,20,42,44] were included in the qualitative synthesis only. Four studies [16,17,18,19] were excluded from the quantitative analysis because the available population data allowed effect estimates to be derived only for the association between mode of delivery and food allergy, but not for exposure to microbiome-modulating medications or the exposure specific data were insufficient for inclusion in the quantitative analysis. Notably, Kvenshagen et al. [18] reported an odds ratio for food allergy in relation to mode of delivery (OR = 0.84), while Alviani et al. [16] provided *p*-values for caeseran section deliveries, showing no statistically significant association for planned C-section (*p* = 0.7775) but a statistically significant for emergency C-section (*p* = 0.048). Although these data indicate a relationship between mode of delivery and food allergy, they could not be incorporated into the meta-analysis focusing on microbiome-modulating medication exposure. Two studies [14,20] were excluded from the quantitative analysis due to insufficient and incomplete data which precluded the calculation of effect estimates. In a prospective birth cohort study, Gao et al. examined Chinese children from Changsha City [14]. The cumulative incidence of food allergy during the first year of life was 22.1%. Several potential risk factors were assessed, including antibiotic exposure during pregnancy and early life. Although these exposures were examined in relation to both food allergy and eczema, a statistically significant association was reported only with eczema. In the Lithuanian birth cohort study, Dubakiene et al. investigated early allergic sensitization and reported an increasing incidence of food allergen senzitization during the first year of life, rising from 1.3% at 6 months to 2.8% at 12 months of age [20]. Maternal factors, including certain maternal diseases and antibiotic use during pregnancy, were not significantly associated with early sensitization to food allergens (*p* > 0.05). Two studies [42,44], which investigated probiotic exposure, were excluded from the quantitative analysis due to differences in study design. While the studies included in the probiotic meta-analysis were randomized double-blind placebo-controlled trials, one of these was a prospective cohort study and another one was a non-treatment controlled trial. Therefore, their results were not considered directly comparable and were included only in the qualitative synthesis. Ching-Wei et al. [42] evaluated the association between maternal probiotic supplementation during pregnancy and the risk of food allergy in children and found no statistically significant association (crude OR: 0.79, 95% CI [0.49, 1.26], aOR: 0.73, 9% CI [0.40, 1.32]). The authors also highlighted that current evidence regarding maternal probiotic supplementation during pregnancy remains limited and that, although probiotics may reduce the risk of eczema, a protective effect against food allergy has not been consistently demonstrated. Dissanayake et al. [44] evaluated the preventive effect of synbiotics and skincare on the development of allergic diseases in infants up to 1 year of age. Synbiotics, which combine probiotics with prebiotic components to enhance microbial activity, were administered either alone or in combination with topical skincare. The study found no evidence that synbiotics, emollient therapy or their combination prevented the development of atopic dermatitis or food allergy during the first year of life.

#### 3.2.2. Quantitative Analysis

A total of 25 studies [3,6,13,21,22,23,24,25,26,27,28,29,30,31,32,33,34,35,36,37,38,39,40,41,43] were included in the quantitative statistical analysis. Of these, 19 studies [3,6,13,21,22,23,24,25,26,27,28,29,30,31,32,33,34,35,36] (5 case–control studies, 3 retrospective studies and 11 prospective birth cohort studies) investigated antibiotic exposure. Exposure to antibiotics was assessed across different time periods, including nine studies that evaluated prenatal exposure, three that assessed postnatal exposure at a single time point, 12 that assessed postnatal exposure multiple times, and one that assessed a different types of antibiotics (Table 1). Several studies contributed data to multiple exposure periods or examined antibiotics alongside other microbiome-modulating medications, such as acid-suppressive agents. Overall, six studies [6,22,28,37,38,39] investigated acid-suppressive medications, including three retrospective and three prospective birth cohort studies with three studies evaluating PPIs and three evaluating H_2_RAs (Table 1). In addition, 3 studies [40,41,43] assessed early-life probiotic exposure. All the three were placebo-controlled studies.

Regarding geographical distribution, from the 25 studies 7 originated from Asia and Australia, 8 from Europe and 10 from the United States. Across studies, data were predominantly collected using administrative or healthcare databases and parent-reported questionnaires (Table 1).

### 3.3. Analyses of Antibiotic Exposure and the Risk of Childhood Food Allergy

#### 3.3.1. Prenatal Antibiotic Exposure

**Figure 2:**
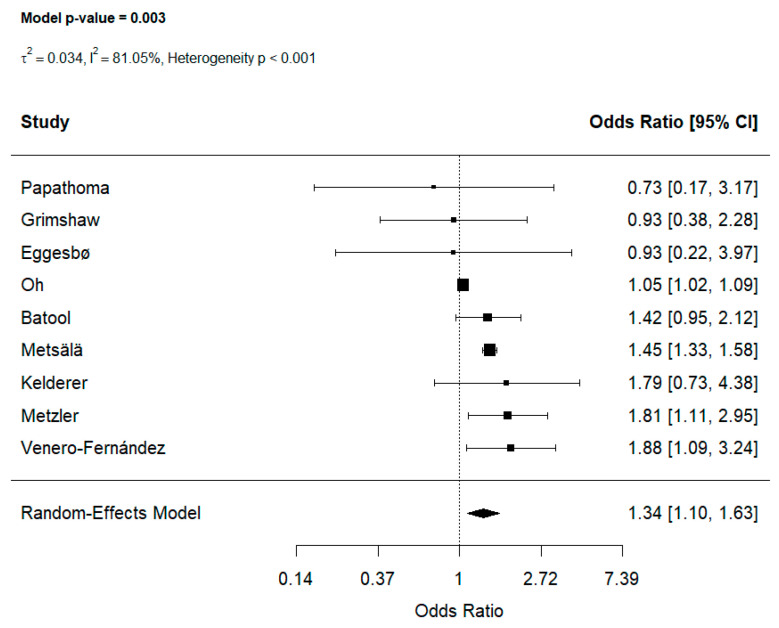
Effect of maternal prenatal antibiotic use on food allergy [22,24,26,29,30,31,32,33,34].

Of the 19 studies investigating antibiotic exposure, data from 9 studies [22,24,26,29,30,31,32,33,34] contributed to the prenatal exposure analyses. Prenatal exposure was associated with increased unadjusted odds of childhood food allergy (OR: 1.34, 95% CI [1.10, 1.63] Figure 2), with substantial heterogeneity across analyses (I^2^ = 81.05%, heterogeneity *p* < 0.001). Leave-one-out sensitivity analyses demonstrated that the pooled effect estimate was not driven by any single study, with ORs remaining consistently elevated and statistically significant across all iterations (range of ORs 1.30–1.46, See Supplementary Figure S1).

Potential publication bias was assessed by funnel plots, which indicated significant asymmetry (Supplementary Figure S2), however, Egger’s test did not indicate publication bias (OR: z = 0.178, *p* = 0.858). To address this bias, trim-and-fill method was performed, and the results remained significant OR: 1.341; 95% CI: [1.104; 1.628].

#### 3.3.2. Postnatal Antibiotic Exposure at a Single Time Point

Among the 19 studies examining antibiotic exposure, three [13,23,25] specifically reported data on postnatal exposure at a single time point. Exposure was linked to higher unadjusted odds of childhood food allergy (OR: 1.85, 95% CI [1.53, 2.23]), with moderate variability across studies (I^2^ = 11.7%, heterogeneity *p* = 0.445). Sensitivity analyses using a leave-one-out approach confirmed that no individual study disproportionately influenced the pooled estimate, with ORs remaining elevated and statistically meaningful throughout all iterations (see Supplementary Figures S3 and S4).

#### 3.3.3. Postnatal Antibiotic Exposure Multiple Time

**Figure 3:**
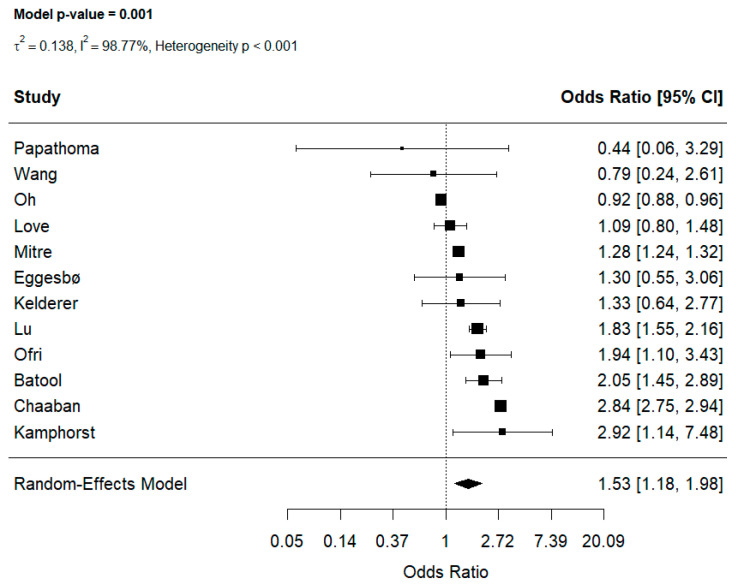
Effect of multiple postnatal antibiotic exposure on food allergy [6,21,23,24,26,27,28,30,32,33,35,36].

A total of 12 studies [6,21,23,24,26,27,28,30,32,33,35,36] provided data on multiple postnatal antibiotic exposure. Repeated exposure was also associated with increased risk of childhood food allergy (OR: 1.53, 95% CI [1.18, 1.98], Figure 3) and there was substantial heterogeneity among studies (I^2^ = 98.77%, heterogeneity *p* < 0.001). Leave-one-out analyses confirmed the robustness of the pooled estimate, with ORs ranging [1.38, 1.60] (Supplementary Figure S5) across all iterations.

Funnel plot inspection suggested potential publication bias, with evident asymmetry (Supplementary Figure S6). Egger’s test confirmed significant bias (OR: z = −0.704, *p* = 0.481). After adjustment using the trim-and-fill method, the pooled estimate remained statistically significant (OR: 1.55; 95% CI: [1.202, 2.011]).

### 3.4. Analyses of Acid-Suppressive Medications (ASMs) and the Risk of Childhood Food Allergy

#### 3.4.1. ASMs

**Figure 4:**
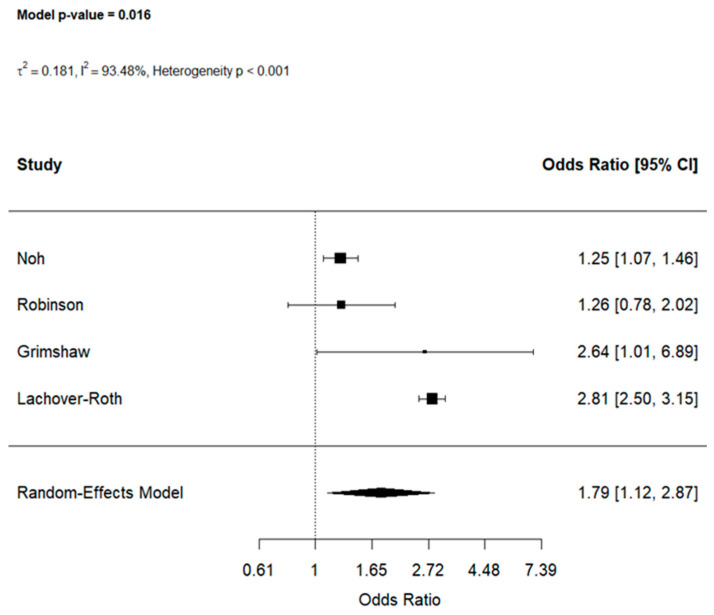
Effect of early-life ASMs exposure on food allergy [29,37,38,39].

A total of four studies [29,37,38,39] investigated ASMs (further not specified) and their association with childhood food allergy. Overall exposure to ASMs was associated with increased unadjusted odds of food allergy (OR: 1.79, 95% CI [1.12, 2.87]) with substantial heterogeneity across studies (I^2^ = 93.48%) (Figure 4). Leave-one-out sensitivity analyses showed that the pooled estimate was not driven by any single study (Supplementary Figure S7).

#### 3.4.2. PPIs and H_2_RAs

**Figure 5:**
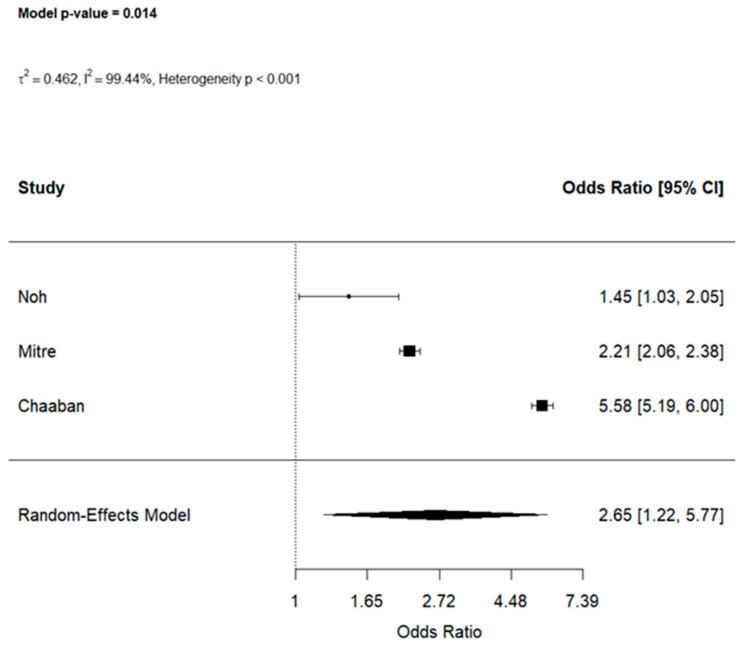
The effects of early life PPIs exposure on food allergy [6,23,37].

When examining specific drug classes, three studies [6,23,37] reported data on PPIs, showing a stronger association (OR: 2.65, 95% CI [1.22, 5.77]) with high heterogeneity (I^2^ = 99.44%, *p* < 0.001), see Figure 5.

Leave-one-out analyses confirmed the robustness of this effect [OR ranging: 1.85, 3.51] (Supplementary Figure S8). The same three studies assessed H_2_RAs which demonstrated a trend toward increased food allergy risk (OR: 2.07, 95% CI [0.96, 4.45], I^2^ = 99.79%, *p* < 0.001) (Supplementary Figures S9 and S10).

### 3.5. Analyses of Probiotic Exposure and the Risk of Childhood Food Allergy

**Figure 6:**
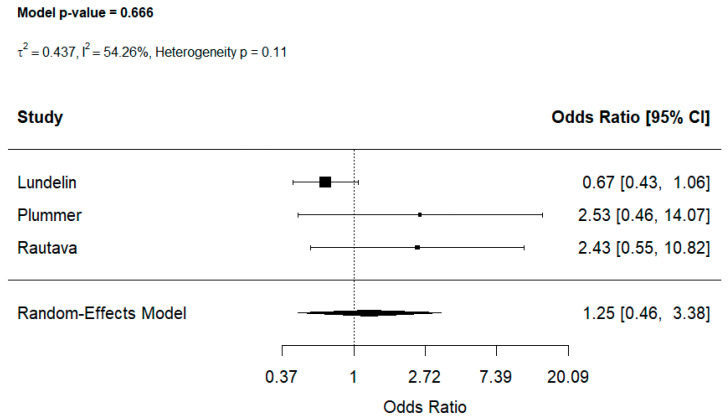
The effects of early life probiotic exposure on food allergy [40,41,43].

Three studies [40,41,43] evaluated the association between probiotic exposure and childhood food allergy. Probiotic use was not significantly associated with the risk of food allergy (OR: 1.25, 95% CI [0.46, 3.38] (I^2^ = 54.26%, *p* = 0.11, Figure 6). Leave-one-out sensitivity analyses confirmed the stability of the findings with pooled ORs ranging [1.00, 2.47], indicating that the overall estimate was not influenced by any single study (Supplementary Figure S11).

### 3.6. Subgroup Analyses

In this meta-analysis, subgroup analyses were conducted to explore potential sources of heterogeneity by stratifying studies according to age at antibiotic exposure, geographic location and type of prescribed antibiotic. Although additional factors, such concomitant medicine use, timing of antibiotic use, mode of delivery, parental history of allergy, and more detailed information on medication exposure supposedely influenced the outcomes, the available data were insufficient to allow meaningful subgroup analyses for these variables. Antiobiotic exposure within the first year of life was associated with increased unadjusted odds of food allergy (OR: 1.41, 95% CI [1.00, 1.98], I^2^ = 99.7%, *p* = 0). Diagnosed between 1 and 3 years of age demonstrated a consistent association with food allergy (OR: 1.46, 95% CI [1.34, 1.58], I^2^ = 0%, *p* = 0.7748), while diagnosed between 3 and 10 years of age was also associated with increased unadjusted odds (OR: 1.84, 95% CI [1.57, 2.15], I^2^ = 0%, *p* = 0.4962). The results are presented in Supplementary Figure S12. Stratification by geographic region, based on 17 studies, showed that the observed associations remained statistically significant across regions, with no clear regional trends identified. Detailed results are summarized in the Table 2 and Supplementary Figure S13.

Finally, subgroup analyses by antibiotic class indicated increased unadjusted odds of food allergy associated with penicillin (OR: 1.19, 95% CI [1.12, 1.27]), cephalosporin (OR: 1.45, 95% CI [1.18, 1.78]) and macrolide exposure (OR: 1.36, 95% CI [1.24, 1.50]) with heterogeneity varying across subgroups. The results are presented in Supplementary Figure S14.

In addition to the predefined subgroup analyses, further subgroup analyses were conducted stratifying studies by study type and food allergy diagnostic method for both antibiotic and acid-suppressive medication exposures. For acid-suppressive medications, subgroup and sensitivity analyses based on diagnostic method could not be performed in a meaningful way, as only a single study reported parent-repoted outcome, while all other studies were based on doctor-diagnosed food allergy, precluding any comparative stratification. For probiotic exposure, such subgroup analyses were not performed due to the inclusion of largely overlapping study sets, which did not allow for meaningful stratification beyond the main analyses. Importantly, the observed associatons remained consistent across all subgroup analyses and no evidence suggested that the overall result were driven by any single study characteristics (see Supplementary Figures S15–S17).

Sensitivity analyses were also performed by excluding studies with less robust outcome definitions. In particular, analyses restricted to doctor-diagnosed food allergy (excluding parent-reported outcomes) showed a slight attenuation of the effect estimate, with the pool unadjusted OR decreasing from 1.45 to 1.38, however heterogeneity remained largely unchanged (see Supplementary Figures S18 and S19).

To further expoler potential sources of heterogeneitiy, separate meta-regression analyses were conducted for each moderator (diagnosis type, study design, continent, and age at diagnosis). None of these variables significantly explained the observed heterogeneity in effect sizes (R^2^ = 0 in all models). This suggests that the substantial heterogeneity is not accounted for by these factors, but may instead be attributable to other methodological differences or study-specific characteristics not reported in the primary literature.

### 3.7. Risk of Bias

Risk of bias for each outcomes were independently assessed by two reviewers (B.T., D.B.) with disagreements resolved by a third person (D.C.). Overall, none of he included studies were judged to be at serious risk of bias and all were retained for the meta-analysis. For maternal prenatal antibiotic exposure, three studies were assessed as low risk of bias and six as moderate risk (Supplementary Figure S20). Among studies examining postnatal antibiotic exposure, five were judged to be at low risk and nine at moderate risk (Supplementary Figure S21). In the acid-suppressive medication group, one of the studies was classified as low risk and five as moderate risk (Supplementary Figure S22). For probiotic interventions, two studies were considered to have low risk and one some concerns (Supplementary Figure S23). Across all studies, bias related to participant selection, exposure classification and selective reporting was generally low while missing data was consistently judged to be at moderate risk. No studies were classified as having a serious risk of bias according to ROBINS-I, however several studies were rated as having moderate risk of bias across key domains, particularly confounding and missing data [15].

### 3.8. Grade of Evidence

The GRADE assessment indicated that the certainty of evidence for an association between maternal prenatal antibiotic exposure and food allergy in childhood was low, reflecting serious concerns regarding risk of bias, incosistency and imprecision. Evidence for postnatal antibiotic exposure whether at a single time point or multiple exposure was also rated as low due to similar limitations. The certainty of evidence for acid-suppressive medication use was low overall while that for H_2_RA exposre was very low primarly owing to substantial imprecision and heterogeneity. Evidence of probiotic supplementation was rated as low with uncertainty remaining due to risk of bias, inconsistency and imprecision across studies (Table 3). However, this analysis was based on a limited number of studies, and the wide confidence intervals indicate substantial uncertainty around the pooled estimate. The outcomes included in this assessment were considered critical for clinical decision-making. Grade of evidence was independently assessed by two reviewers (T.K., D.B.) with disagreements resolved by a third person (B.T.).

## 4. Discussion

Our systematic review and meta-analysis investigated the association between prenatal and early-life exposure to microbiome-modulating medications—including antibiotics, acid-suppressive medications and probiotics—and the risk of food allergy in children. Prenatal antibiotic exposure was consistently linked to higher unadjusted odds of developing childhood food allergy (OR: 1.34, 95% CI [1.10–1.63]), while postnatal antibiotic exposure, both at a single time point and multiple exposure, also conferred elevated risk (ORs: 1.85 and 1.53 respectively). Acid-suppressive medications, particularly PPIs demonstrated a more pronounced association (OR: 2.65, 95% CI [1.22, 5.77]), whereas H_2_RAs showed only a trend toward elevated risk but with very low certainty. Probiotic supplementation was not significantly associated with decreased risk of developing food allergy. The pooled analysis using a random-effects model showed an unadjusted odds ratio of 1.25 (95% CI [0.46, 3.38], indicating no clear evidence of either a protective or a harmful effect.

The observed associations may be explained by disruptions of the developing gut microbiome. Antibiotic-induced dysbiosis may interfere with the normal establishment of immune tolerance, while acid-suppressive medications could alter protein digestion and microbial composition, leading to enhanced immune responsiveness to dietary antigens [45]. The findings related to probiotic exposure should be interpreted with caution. Only a small number of studies were available for this analysis and the wide confidence intervals around the pooled estimate indicate substantial uncertainty. As a result, the current evidence base is limited and does not allow for firm conclusions regarding either a protective or a harmful effect. Rather than indicating a true absence of association, these results should be considered exploratory and highlight the need for further well-designed studies, particularly randomized controlled trials, to clarify the potential role of probiotics in the prevention of food allergy.

Previous systematic reviews have generally focused on broader allergic outcomes, such as asthma, eczema or atopic sensitization, rather than food allergy specifically [8,9,10]. Consequently, these analyses often included a larger number of studies but evaluated heterogeneous outcomes. Our findings are broadly consistent with earlier evidence suggesting that early-life antibiotic exposure and acid-suppressive medication use may increase the risk of allergic diseases [11,12]. However, by focusing exclusively on food allergy as the outcome, the present study provides a more specific estimate of the relationship between microbiome-modulating medications and this clinically relevant condition. Moreover, to our knowledge, no previous meta-analysis has specifically evaluated probiotic exposure in relation to food allergy alone, making this analysis a novel contribution to the literature.

Emerging evidence highlights the central role of the gut–immune–brain axis as a mechanistic framework linking microbiome perturbations to immune dysregulation. This bidirectional system integrates microbial metabolites, immune signaling pathways and neuroendocrine responses, thereby influencing both immune and neurological homeostasis. Disruption of microbial composition and barrier function—particularly during early life—may impair immune education and predispose individuals to aberrant immune responses, including allergic diseases [46].

Early life represents a critical window for microbiome establishment and immune maturation. The development of a balanced microbial community, including keystone taxa such as Bifidobacterium species, is closely linked to the maturation of adaptive immunity and lymphoid tissue architecture. These taxa have also been associated with improved vaccine responses, suggesting that microbiome composition may influence broader aspects of immune competence beyond allergy risk. However, antibiotic exposure is highly prevalent in early childhood and may disrupt the balance of the gut microbiome. While antibiotics remain essential for the treatment of bacterial infections, these observations highlight the importance of optimizing their use to minimise unintended microbiome disruption and its potential long-term consequences [46,47].

Importantly, microbiome-drug interactions are not limited to antibiotics. Increasing evidence suggests that non-antibiotic medications, including PPIs, antipsychotics and metformin, may exert microbiotoxic effects by altering microbial diversity and composition. PPIs have been associated with reduced microbial diversity and an increased susceptibility to enteric infections, indicating impaired colonisation resistance. Similarly, experimental studies have demonstrated that antipsychotics such as aripiprazole can inhibit microbial growth. Moreover, clinical data link these agents to metabolic disturbances, potentially mediated through the microbiome alterations. Metformin has been associated with gastrointestinal side effects and changes in specific microbial taxa, some of which have been linked to adverse cardiometabolic outcomes. These findings support the concept that commonly used medications may inadvertently disrupt host –microbiome homeostasis [47].

Several methodological strengths enhance the reliability of these findings. The review was conducted following PRISMA guidelines, with protocol prospectively registered in PROSPERO, specifying the research question, eligibility criteria, population, comparator and outcomes. We carried out the systematic review and meta-analysis as outlined in the original protocol, making no alterations to the objectives, eligibility criteria, search strategy or methods. All analyses were performed strictly according to the registered protocol, minimizing selective reporting. The certainty of evidence was systematically assessed using GRADE, providing a structured evaluation of methodological quality. These features ensured transparency, reproducibility and a robust framework for interpreting the pooled results.

Nonetheless, important limitations must be acknowledged. The high heterogeneity observed in several studies may reflect differences in study design, exposure assessment, outcome definitions and population characteristics, which may influence pooled estimates. In addition, variability in diagnostic criteria for food allergy and the lack of individual patient data further limit comparability across studies. The number of eligible studies for certain exposures—particularly probiotics and acid-suppressive medications—was relatively small, resulting in wide confidence intervals and reduced statistical power. Accordingly, the overall certainty of evidence was low for most exposures and very low for H_2_RAs according to GRADE analyses. Importantly, the certainty of evidence for most outcomes was rated as low or very low according to GRADE, reflecting concerns related to heterogeneity, risk of bias, and imprecision. Therefore, the observed associations should not be interpreted as evidence of causality but rather as indicative of potential relationships that warrant further investigation.

From a conceptual perspective, most of the available evidence derives from observational studies, limiting causal inference and residual confounding cannot be fully excluded. Residual confounding represents an important limitation of this meta-analysis. Unfortunately, not enough included studies reported adjusted effect estimates and the type and number of confounders considered varied substantially across studies. In particular, confounding by indication is a major concern in the context of medication exposure. For example, antibiotics or acid-suppressive medications may be prescribed to children with underlying conditions, infections or early symptoms that themselves could be associated with an increased risk of allergic disease. As a result, the observed associations may partly reflect differences in baseline risk rather than a direct causal effect of the medications. In addition, several important confounders, such as parental history of allergy, mode of delivery, breastfeeding and other environmental or clinical factors, were not consistently accounted for across studies. Therefore, residual confounding cannot be excluded and should be considered when interpreting the pooled estimates.

An additional important consideration is the variation in bias structures across the different study designs included in this meta-analysis. Most of the evidence was derived from observational studies, including prospective and retrospective cohort studies as well as case–control designs. Prospective cohort studies allow for a clearer temporal relationship between exposure and outcome and are less prone to recall bias, however they remain susceptible to residual confounding. Retrospective and database-based studies may introduce misclassification bias due to reliance on administrative codes and limited clinical detail regarding both exposure and outcome definitions. Case–control studies are more vulnerable to recall and selection bias, which may influence the observed associations. In contrast, the randomized controlled trials included in the probiotic analysis provide a higher level of casual inference but are limited by smaller sample sizes and potential issues with generalizability. These differences in bias structures may have contributed to the observed heterogeneity and should be considered when interpreting the pooled effect estimates.

Although no studies were classified as having a serious risk of bias, this finding should be interpreted with caution. Most included studies were observational and therefore inherently susceptible to bias, particularly due to confounding and exposure misclassification. Several studies were rated as having moderate risk of bias across key domains, which may have influenced the observed associations. In particular, bias due to confounding is difficult to fully address in non-randomized studies of medication exposure and the ROBINS-I tool may not fully capture the complexity of these biases. Therefore, the overall risk-of-bias assessment should not be interpreted as indicating a low likelihood of bias, but rather as reflecting relative grading within the constraints of the inherent limitations of observational study designs.

Publication bias also represents a potential limitation of the present analysis, and it yielded somewhat inconsistent findings. Visual inspection of funnel plots suggested asymmetry in several analyses, whereas Egger’s test did not consistently indicate statistically significant bias. This apparent discrepancy may be explained by limited statistical power of formal tests for publication bias, particularly when the number of included studies is small. In such cases, non-significant results from Egger’s test do not necessarily exclude the presence of publication bias. Moreover, funnel plot asymmetry may arise not only from publication bias but also from other sources, including heterogeneity, differences in study size or methodological variability across studies. Although in cases of antibiotic use, the trim-and-fill procedure did not meaningfully alter the pooled effect size, suggesting no clear evidence of funnel plot asymmetry, the limited sensitivity of this method—particularly in the presence of a small number of studies or substantial heterogeneity—means that publication bias cannot be definitively ruled out. Therefore, the results of publication bias assessments should be interpreted with caution. Given the substantial heterogeneity observed in several analyses, the observed asymmetry may also reflect genuine differences between studies rather than selective publication.

Further research should aim to address these limitations through the establishment of large-scale prospective registries and more standardized and detailed reporting of study characteristics, including diagnostic criteria and timing of exposure. Such efforts would improve comparability across studies and strengthen the evidence base regarding the relationship between microbiome-modulating medications and food allergy risk.

From a clinical perspective, these findings emphasize the importance of the careful use of microbiome-modulating medications during pregnancy and in the early years of life. In light of the growing clinical interest in probiotic-based prevention strategies, the inconclusive findings of this meta-analysis suggest a substantial knowledge gap rather than an absence of biological relevance. Therefore, future high-quality randomised controlled trials are required, taking into account strain specificity, the timing of exposure, and clinically meaningful endpoints, before firm preventive recommendations can be established.

In summary, this study provides the most comprehensive evidence to date on maternal and early-life microbiome modulation and childhood food allergy. High-quality prospective cohorts and randomized trials are urgently needed to clarify causality and inform preventive strategies.

## 5. Conclusions

This meta-analysis suggests that prenatal and early-life exposure to antibiotics and acid-suppressive medications may be associated with an increased risk of food allergy in children. However, the overall certainty of evidence was low or very low for most outcomes, therefore these findings should be interpreted with caution.

Probiotic supplementation was not associated with a statistically significant effect, however given the limited number of studies and the wide confidence intervals, this finding should be interpreted with caution and considered exploratory.

Overall, the results indicate potential associations rather than causal relationships and further high-quality studies are needed to confirm these findings and clarify the underlying mechanisms.

## Figures and Tables

**Table 1:** Characteristics of included studies.

First Author, Year of Publication, Country	Age at the Time of Diagnosis	Intervention Type	Study Type	Outcome: Unadjusted Odds Ratio for Food Allergy (as Defined in Indvidual Studies)
Batool, 2016 [32], Canada (Hamilton, Burlington)	first year of life	prenatal ABs and postnatal ABs multiple times	prospective cohort study	OR_prenatal_: 1.42 95% CI [0.95, 2.12] OR_postnatal_: 2.05 95% [1.45, 2.89]
Chaaban, 2025 [23], USA	during first year	postnatal ABs at a single point and multiple times	retrospective cohort study	OR_single_: 1.92 95% CI [1.88, 1.97] OR_multiple_: 2.84 95% CI [2.75, 2.94]
Eggesbø, 2003 [30], Norway (Oslo)	until 24 months	prenatal ABs and postnatal ABs multiple times	prospective cohort study	OR_prenatal_: 0.93 95% CI [0.38, 2.28] OR_postnatal_: 1.30 95% CI [0.55, 3.06]
Grimshaw, 2016 [29], UK	until 2 years	prenatal ABs	prospective cohort study	OR: 0.93 95% CI [0.38, 2.28]
Hirsch, 2016 [3], USA (Pennsylvania)	until 7 years old	different type of antibiotics	case–control study	OR_Penicillin_: 1.59 95% CI [1.10, 2.29] OR_Cephalosporin:_ 1.77 95% CI [0.97, 3.24] OR_Macrolide_: 1.51 95% CI [0.60, 3.81]
Love, 2016 [21], USA	until 12 months	postnatal ABs multiple times	case–control study	OR: 1.09 95% CI [0.08, 1.48]
Lu, 2024 [27], China (Charsa)	3–6 years	postnatal ABs multiple times	prospective cohort study	OR: 1.83 95% CI [1.55, 2.16]
Kamphorst, 2021 [28], Netherlands	4–6 years	postnatal ABs multiple times	prospective cohort study	OR: 2.92 95% CI [1.14, 7.48]
Karpa, 2012 [13], USA	1.5 years	postnatal ABs at a single point time	case–control study	OR: 1.35 95% CI [0.68, 2.68]
Kelderer, 2022 [24], Sweden	during first 18 months	prenatal ABs and postnatal ABs multiple times	prospective birth cohort study	OR_prenatal_: 1.79 95% CI [0.73, 4.38] OR_postnatal_: 1.33 95% CI [0.64, 2.77]
Metsälä, 2013 [31], Finland	until 2 years	prenatal ABs	case–control study	OR: 1.45 95% CI [1.33, 1.58]
Metzler, 2019 [22], Austria, Finland, France, Germany, Switzerland	up to 2 years	prenatal ABs	prospective cohort study	OR: 1.81 95% CI [1.11, 2.95]
Mitre, 2018 [6], USA	during first 6 months	postnatal ABs multiple times	retrospective cohort study	OR: 1.28 95% CI [1.24, 1.32]
Ofri, 2025 [36], Israel	first 60 days	postnatal ABs multiple times	retrospective cohort study	OR: 1.94 95% CI [1.10, 3.43]
Oh, 2024 [33], South Korea	until 6 months	prenatal ABs and postnatal ABs multiple times	prospective cohort study	OR_prenatal_:1.05 95% CI [1.02, 1.09] OR_postnatal_: 0.92 95% CI [0.88, 0.96]
Papathoma, 2016 [26], Greece	until 36 months	prenatal ABs and postnatal ABs multiple times	prospective cohort study	OR_prenatal_: 0.73 95% CI [0.17, 3.17] OR_postnatal_: 0.44 95% CI [0.66, 3.29]
Renz-Polster, 2005 [25], USA (Oregon)	3–10 years	postnatal ABs at a single point time	prospective cohort study	OR: 1.34 95% CI [0.54, 3.31]
Venero-Fernández, 2018 [34], Cuba (Havana)	first 3 years of life	prenatal ABs	prospective cohort study	OR: 1.88 95% CI [1.09, 3.24]
Wang, 2022 [35], China (Peking)	until 6 months	postnatal ABs multiple times	case–control study	OR: 0.79 95% CI [0.24, 2.61]
Chaaban, 2025 [23], USA	first year of life	H_2_RAs and PPIs	retrospective cohort study	OR_H2RAs_: 4.35 95% CI [4.14, 4.58] OR_PPIs_: 5.58 95% CI [5.19, 6.00]
Grimshaw, 2016 [29], UK	until 2 years	ASMs	prospective cohort study	OR: 2.64 95% CI [1.01, 6.89]
Lachover-Roth, 2025 [39], Israel	first 6 months of life	ASMs	retrospective cohort study	OR: 2.81 95% CI [2.50, 3.15]
Mitre, 2018 [6], USA	first 6 months of life	H_2_RAs and PPIs	retrospective cohort study	OR_H2RAs_: 1.74 95% CI [1.67, 1.81] OR_PPIs_: 2.21 95% CI [2.06, 2.38]
Noh, 2023 [37], South Korea	first 6 months of life	ASMs, H_2_RAs, PPIs	prospective cohort study	OR_ASMs_: 1.25 95% CI [1.07, 1.46] OR_H2RAs_: 1.16 95% CI [0.98, 1.37] OR_PPIs_: 1.45 95% CI [1.03, 2.05]
Robinson, 2022 [38], USA	first year of life	ASMs	prospective cohort study	OR: 1.26 95% CI [0.78, 2.02]
Lundelin, 2016 [40], Finland (Turku)	until 10 years	probiotics	placebo-control study	OR: 0.67 95% CI [0.43, 1.05]
Plummer, 2019 [41], Australia (Victoria)	first 2 years of life	probiotics	placebo-control study	OR: 2.53 95% CI [0.46, 13.99]
Rautava, 2002 [43], Finland (Helsinki)	until 2 years	probiotics	placebo-control study	OR: 2.43 95% CI [0.55, 10.78]

**Table 2:** Stratification by geographic region according to postnatal antibiotic exposure.

Stratification by Geographic Region According to Postnatal Antibiotic Exposure			
Continent	USA	Europe	Asia
Number of studies	7	6	4
ORs with 95% CIs	1.57 [1.16, 2.12]	1.45 [1.33, 1.58]	1.34 [0.86, 2.10]

**Table 3:** GRADE summary of findings for each intervention.

Exposure	No. of Studies	Risk of Bias	Inconsistency	Indirectness	Imprecision	Certainty	Relative Effect (Random Effects OR, 95% CI)
**Prenatal antibiotic exposure**	9	Serious	Serious	Not serious	Serious	⨁⨁◯◯ Low	**1.34 (1.10–1.63)**
**Postnatal antibiotic exposure (single time point)**	3	Serious	Serious	Not serious	Serious	⨁⨁◯◯ Low	**1.85 (1.53–2.23)**
**Postnatal antibiotic exposure (multiple time points)**	12	Serious	Serious	Not serious	Serious	⨁⨁◯◯ Low	**1.53 (1.18–1.98)**
**Acid-suppressive medication (overall)**	4	Serious	Serious	Not serious	Serious	⨁⨁◯◯ Low	**1.79 (1.12–2.87)**
Proton pump inhibitors (PPI)	3	Serious	Serious	Not serious	Serious	⨁⨁◯◯ Low	**2.65 (1.22–5.77)**
H_2_ receptor antagonists (H_2_RA)	3	Serious	Serious	Not serious	Serious	⨁◯◯◯ Very low	**2.07 (0.96–4.45)**
**Probiotic exposure**	3	Not serious	Not serious	Not serious	Serious	⨁⨁◯◯ Low	**1.25 (0.46–3.38)**

## Data Availability

Data extracted from the included studies that are not reported in the article or in the supporting information can be obtained from the authors.

## Supplementary Materials

The following supporting information can be downloaded at: https://www.mdpi.com/article/10.3390/jcm15083086/s1, File S1: Supplementary figures: Figure S1: Leave-one-out meta-analysis for prenatal antibiotic exposure with 95% CIs, Figure S2: The results of prenatal antibiotic exposure remain significant according to Egger’s test and trim-and-fill method, Figures S3 and S4: Postnatal antibiotic exposure at a single point time with 95% CIs and leave-one-out meta-analysis, Figure S5: Postnatal antibiotic exposure multiple time with 95% CIs and leave-one-out meta-analysis, Figure S6: The results of prenatal antibiotic exposure remain significant according to Egger’s test and trim-and-fill method, Figure S7: Leave-one-out meta-analysis for ASMs exposure, Figure S8: Forest plot of individual study ORs for PPIs exposure with 95% CIs and leave-one-out meta-analysis, Figures S9 and S10: Forest plot of individual study ORs for H2RAs exposure with 95% CIs and leave-one-out meta-analysis, Figure S11: Leave-one-out meta-analysis for probiotic exposure with 95% CIs, Figure S12: Subgroup analysis of the association between antibiotic exposure and food allergy according to age at diagnosed, Figure S13: Subgroup analysis of the association between antibiotic exposure and food allergy by geographic region, Figure S14: Subgroup analysis of the association between antibiotic exposure and food allergy according to antibiotic classes, Figure S15: Subgroup analysis of the association between study type and food allergy according to antibiotic exposure, Figure S16: Subgroup analysis of the association between food allergy diagnostic method and food allergy according to antibiotic exposure, Figure S17: Subgroup analysis of the association study type and food allergy according to acid-suppressive medications exposure, Figure S18: Subgroup analysis of the association study type and food allergy according to acid-suppressive medications exposure, Figure S19: Subgroup analysis of the association study type and food allergy according to acid-suppressive medications exposure, Figure S20: Risk of bias of prenatal antibiotic exposure, Figure S21: Risk of bias of postnatal antibiotic exposure, Figure S22: Risk of bias of acid-suppressive medication exposure, Figure S23: Risk of bias of probiotic exposure; File S2: PRISMA checklist [48].

## Abbreviations

ABs	antibiotics
aOR	adjusted odds ratios
ASMs	acid-suppressive medications
CENTRAL	Cochrane Central Register of Controlled Trials
CIs	confidence intervals
GDT	Guideline Development Tool
GRADE	Grading of Recommendations Assessment, Development and Evaluation
H_2_RAs	histamine-2-receptor antagonists
I^2^	I-squared
ORs	odds ratios
PICOS	patients, intervention, control, outcome, study design
PPIs	proton pump inhibitors
PRISMA	Preferred Reporting Items for Systematic Reviews and Meta-Analyses
PROSPERO	Prospective Register of Systematic Reviews
ROBINS-I	Risk of Bias In Non-Randomized Studies—of Interventions
τ^2^	tau-squared
