# Supplementary material for: Prenatal and Early-Life Exposure to Microbiome-Modulating Medications and the Risk of Childhood Food Allergy: A Systematic Review and Meta-Analysis
Source: J Clin Med. 2026 Apr 17;15(8):3086. doi: 10.3390/jcm15083086 (PMC13117669; doi:10.3390/jcm15083086)
Supplement: Supplementary file 1 [file jcm-15-03086-s001.zip › jcm-4247254-supplementary/jcm-4247254-File S1.pdf]

Supplementary Information

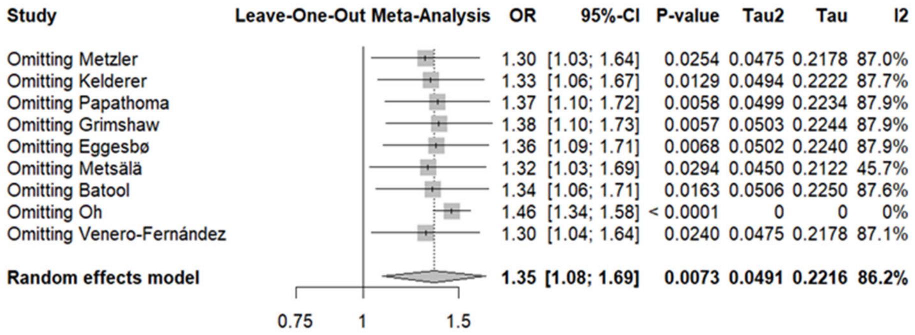

Supplementary Figure S1: Leave-one-out meta-analysis for prenatal antibiotic exposure with 95% CIs.

```
--- Publication Bias Analysis ---
Egger's Regression Test: z = 0.178 , p = 0.858
(ok) Nincs jelentős aszimmetria (p >= 0.05).
---------------------------------

> print(final_bias_table)
                    Modell    OR CI_lower CI_upper P_value Missing_K
1     Eredeti (Observed) 1.341    1.104    1.628 0.00307         0
2 Trim & Fill (Adjusted) 1.341    1.104    1.628 0.00307         0
>
```

Supplementary Figure S2: The results of prenatal antibiotic exposure remain significant according to Egger's test and trim-and-fill method.

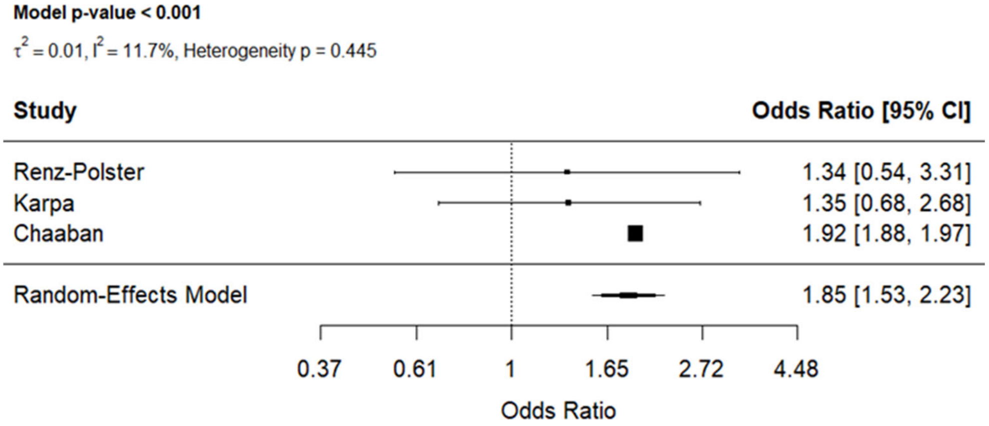

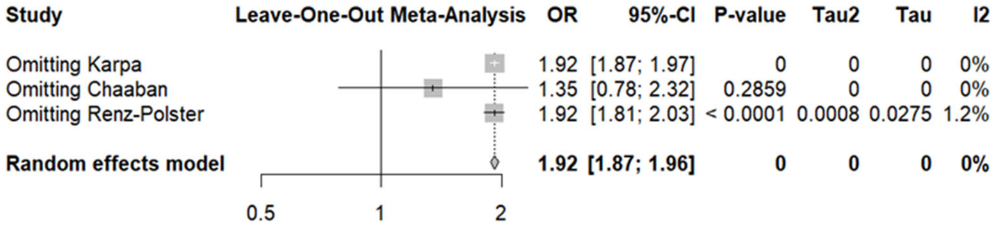

Supplementary Figure S3-S4: Postnatal antibiotic exposure at a single point time with 95% CIs and leave-one-out meta-analysis.

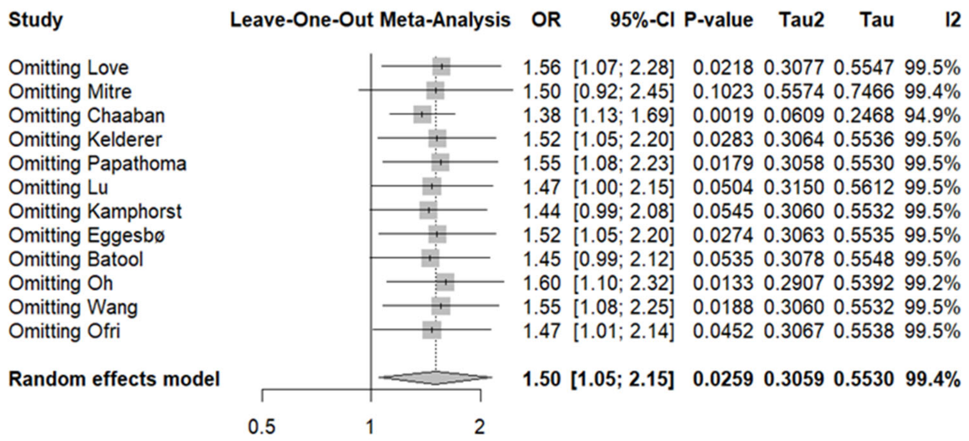

Supplementary Figure S5: Postnatal antibiotic exposure multiple time with 95% CIs and leave-one-out meta-analysis.

```
--- Publication Bias Analysis ---
Egger's Regression Test: z = -0.704 , p = 0.481
(ok) Nincs jelentős aszimmetria (p >= 0.05).
---------------------------------

> print(final_bias_table)
                 Modell    OR CI_lower CI_upper P_value Missing_K
1     Eredeti (Observed) 1.527    1.178    1.978 0.00137         0
2 Trim & Fill (Adjusted) 1.555    1.202    2.011 < 0.001         1
> |
```

Supplementary Figure S6: The results of prenatal antibiotic exposure remain significant according to Egger’s test and trim-and-fill method.

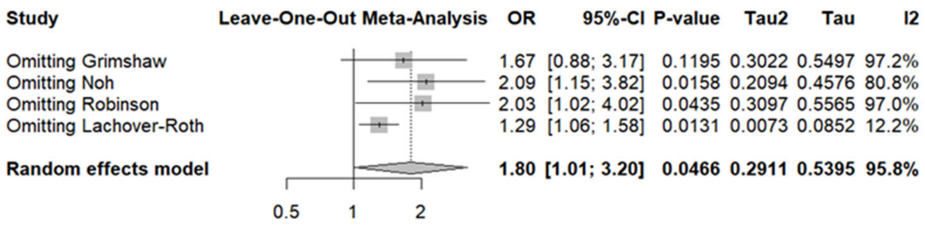

Supplementary Figure S7: Leave-one-out meta-analysis for ASMs exposure.

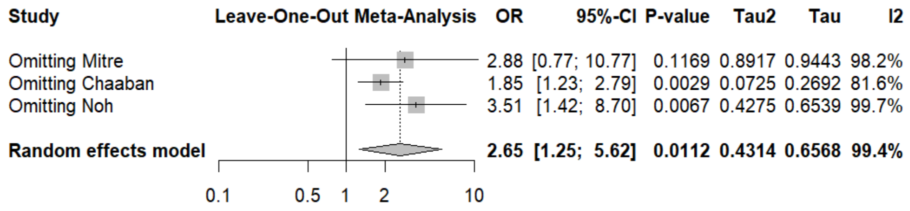

Supplementary Figure S8: Forest plot of individual study unadjusted ORs for PPIs exposure with 95% CIs and leave-one-out meta-analysis

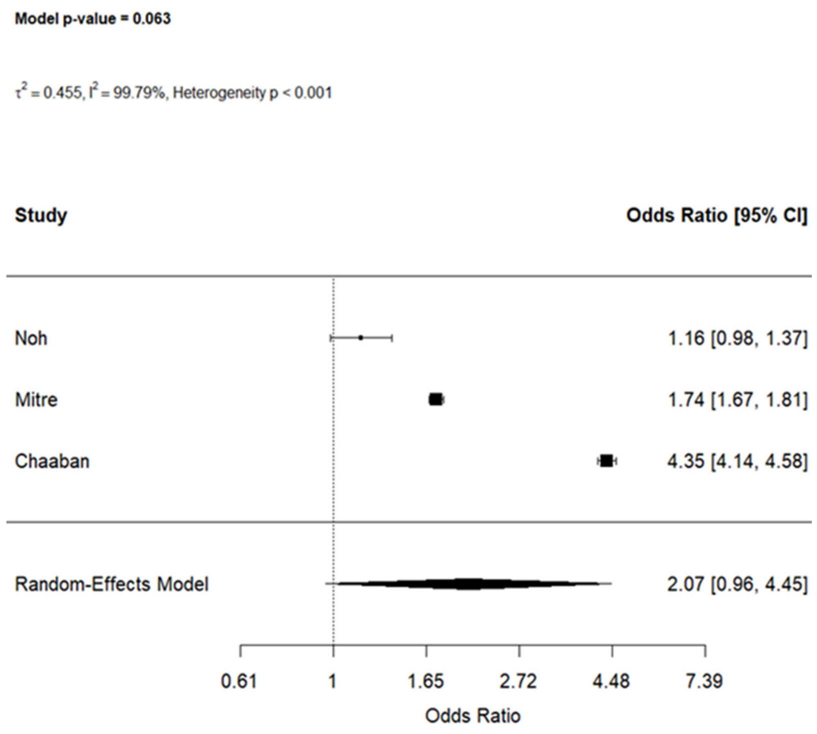

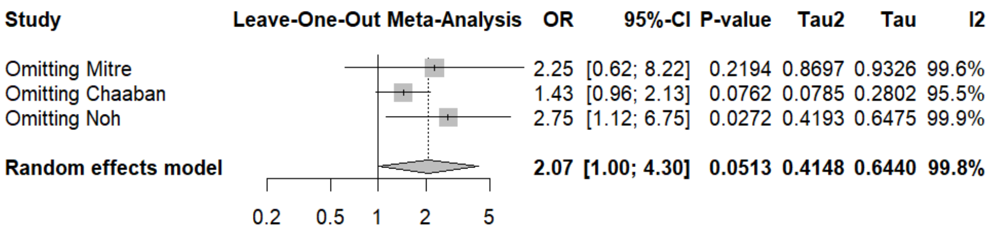

Supplementary Figure S9-S10: Forest plot of individual study unadjusted ORs for H2RAs exposure with 95% CIs and leave-one-out meta-analysis.

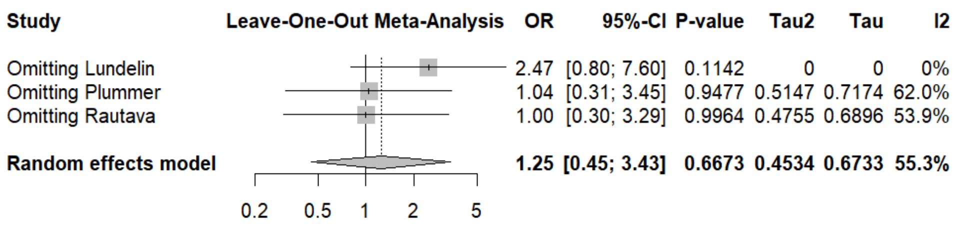

Supplementary Figure S11: Leave-one-out meta-analysis for probiotic exposure with 95% CIs.

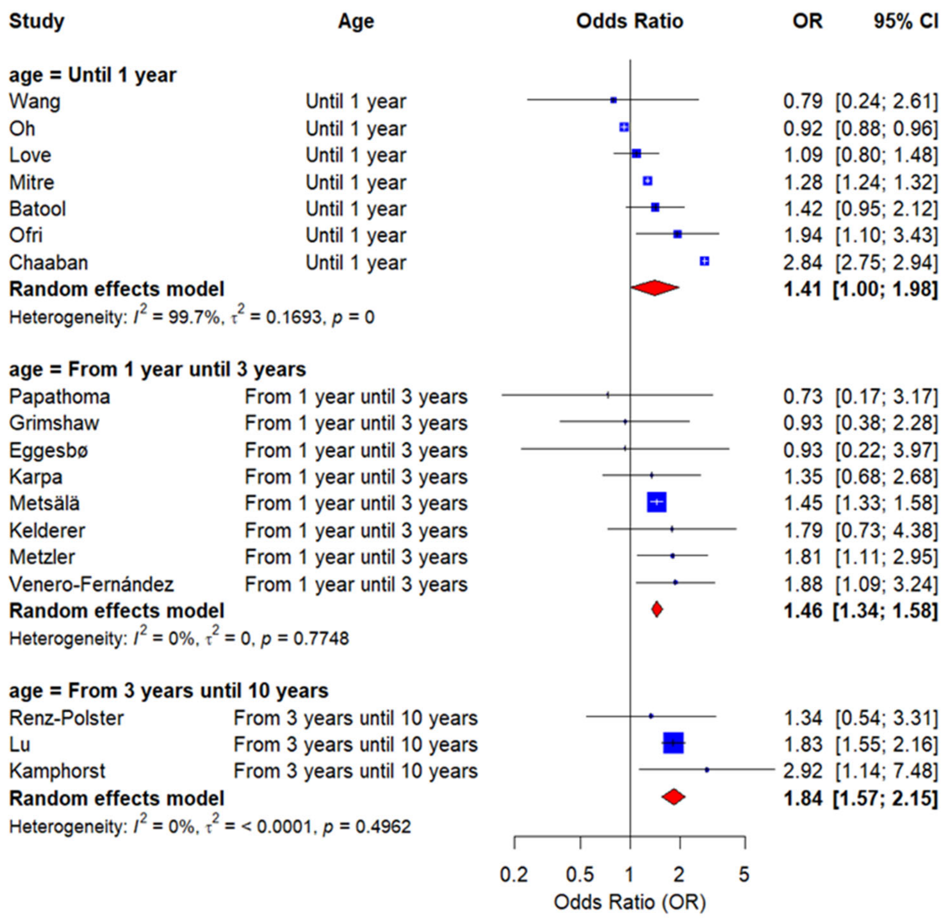

Supplementary Figure S12: Subgroup analysis of the association between antibiotic exposure and food allergy according to age at diagnosed.

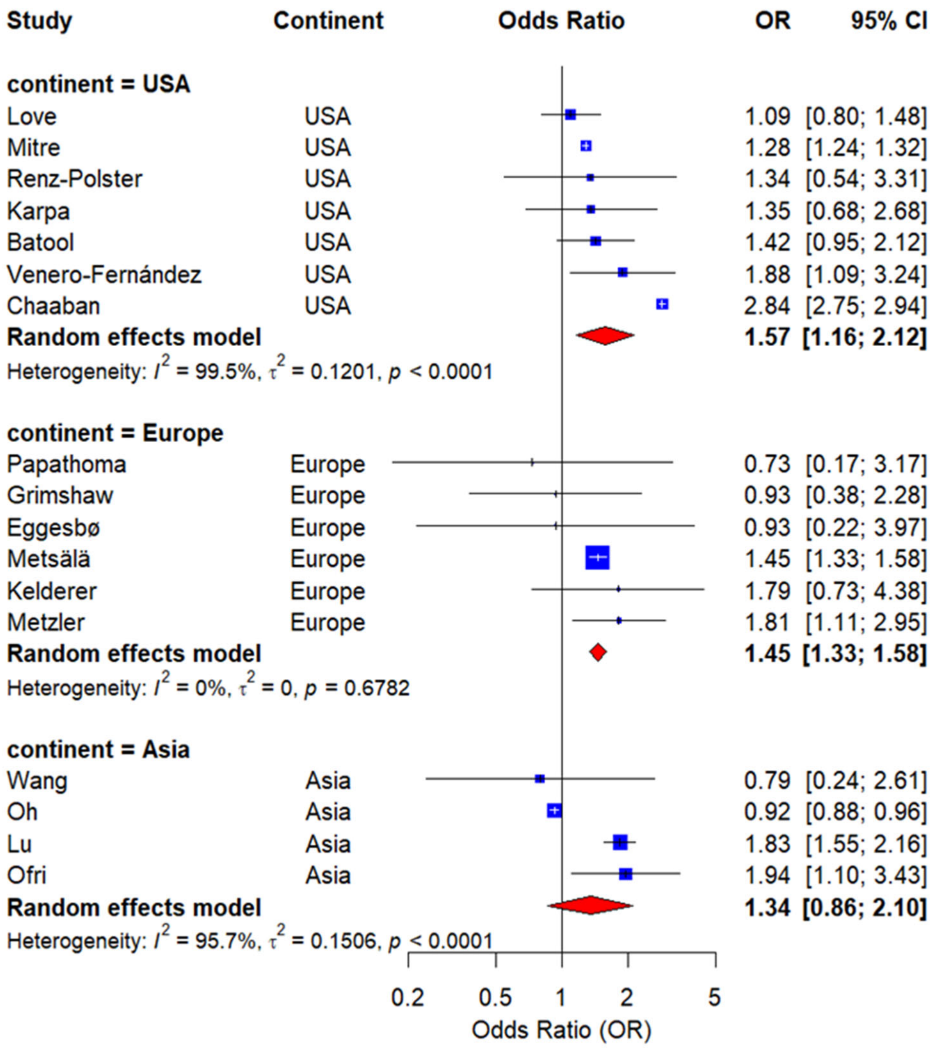

Supplementary Figure S13: Subgroup analysis of the association between antibiotic exposure and food allergy by geographic region

.

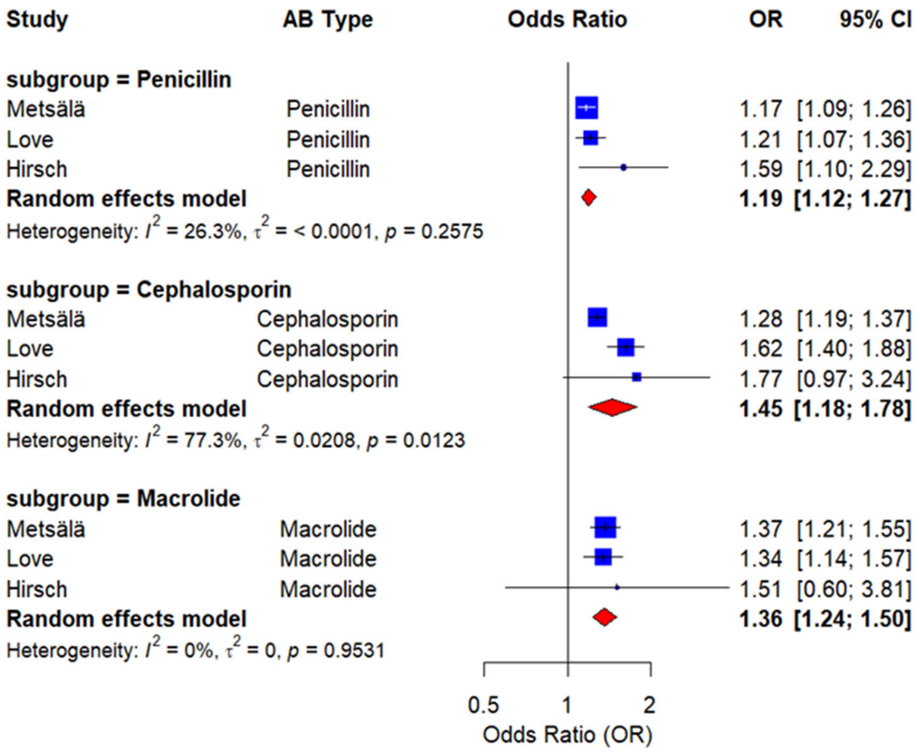

Supplementary Figure S14: Subgroup analysis of the association between antibiotic exposure and food allergy according to atibiotic classes.

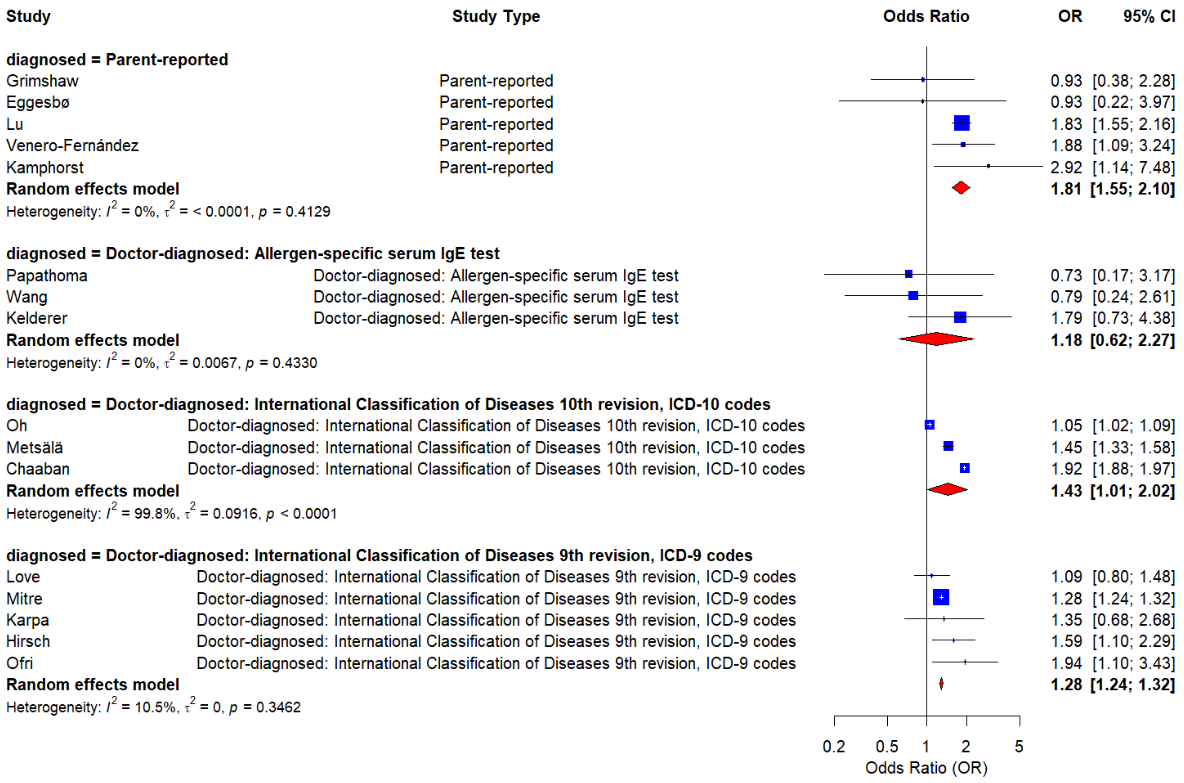

Supplementary Figure S15: Subgroup analysis of the association between study type and food allergy according to antibiotic exposure.

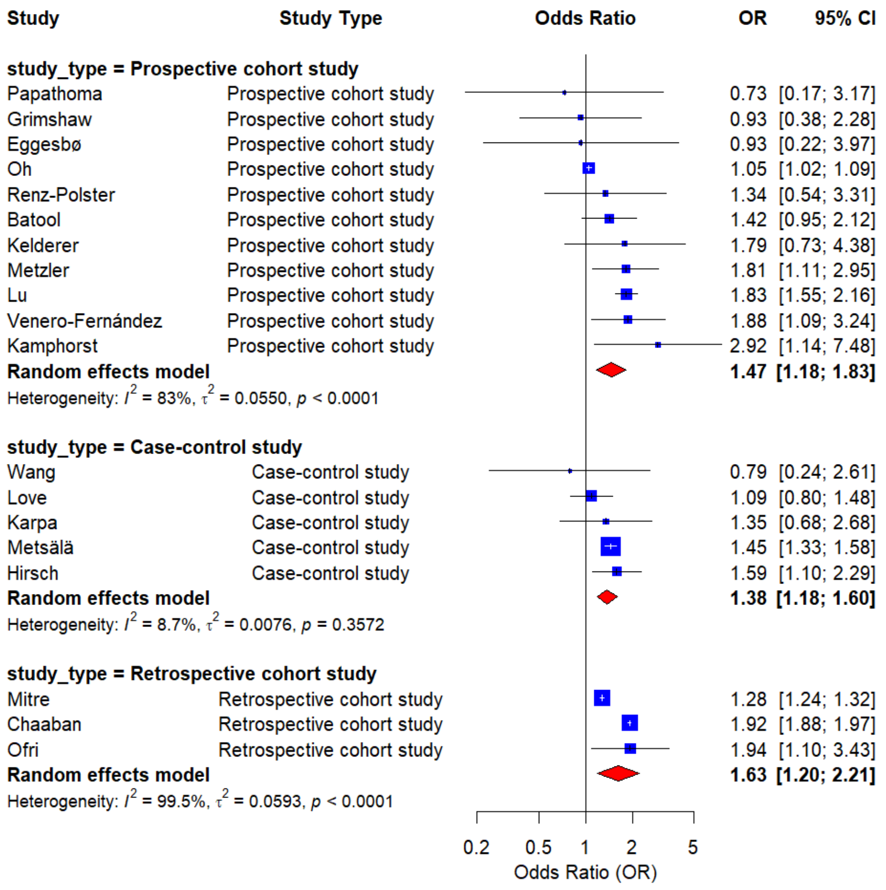

Supplementary Figure S16: Subgroup analysis of the association between food allergy diagnostic method and food allergy according to antibiotic exposure.

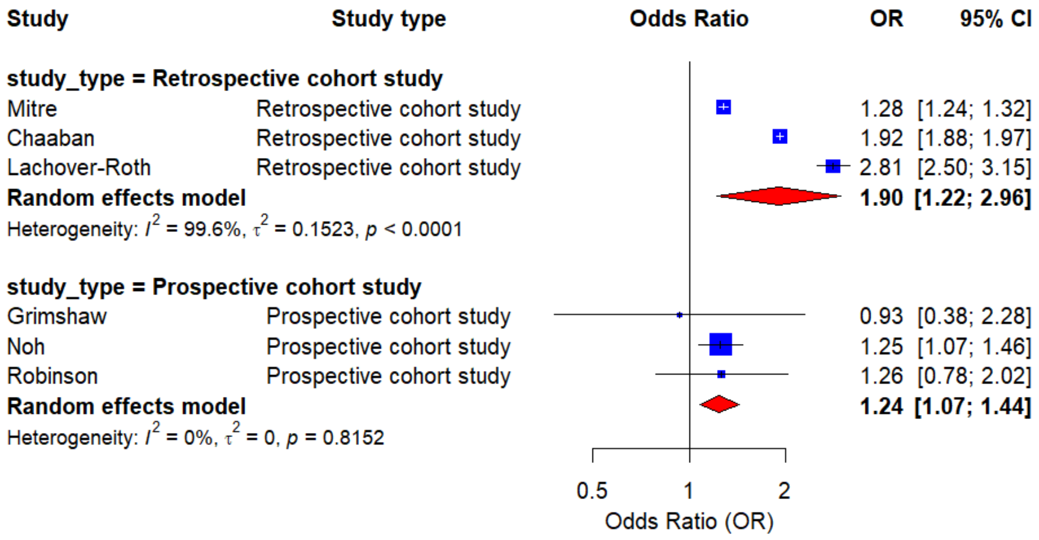

Supplementary Figure S17: Subgroup analysis of the association study type and food allergy according to acid-suppressive medications exposure.

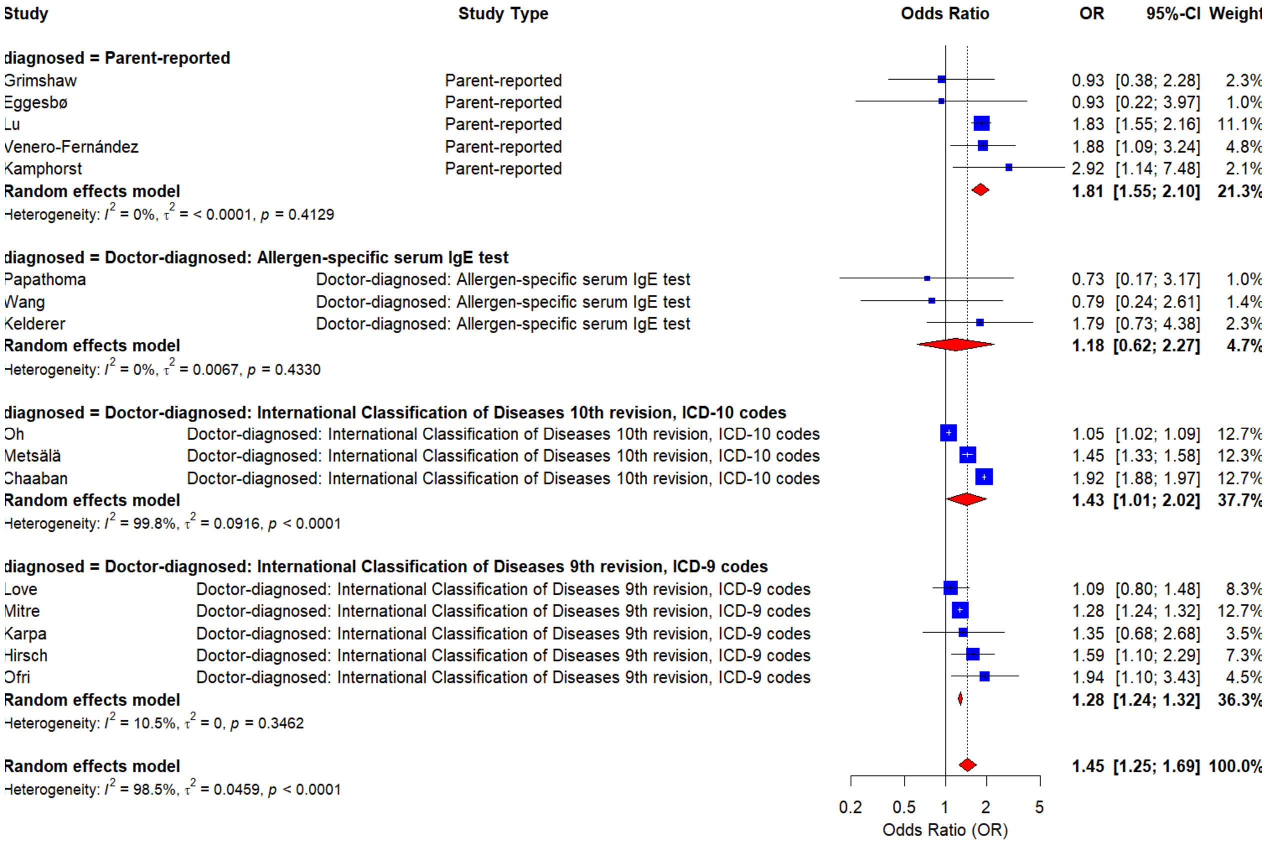

Supplementary Figure S18: Subgroup analysis of the association study type and food allergy according to acid-suppressive medications exposure.

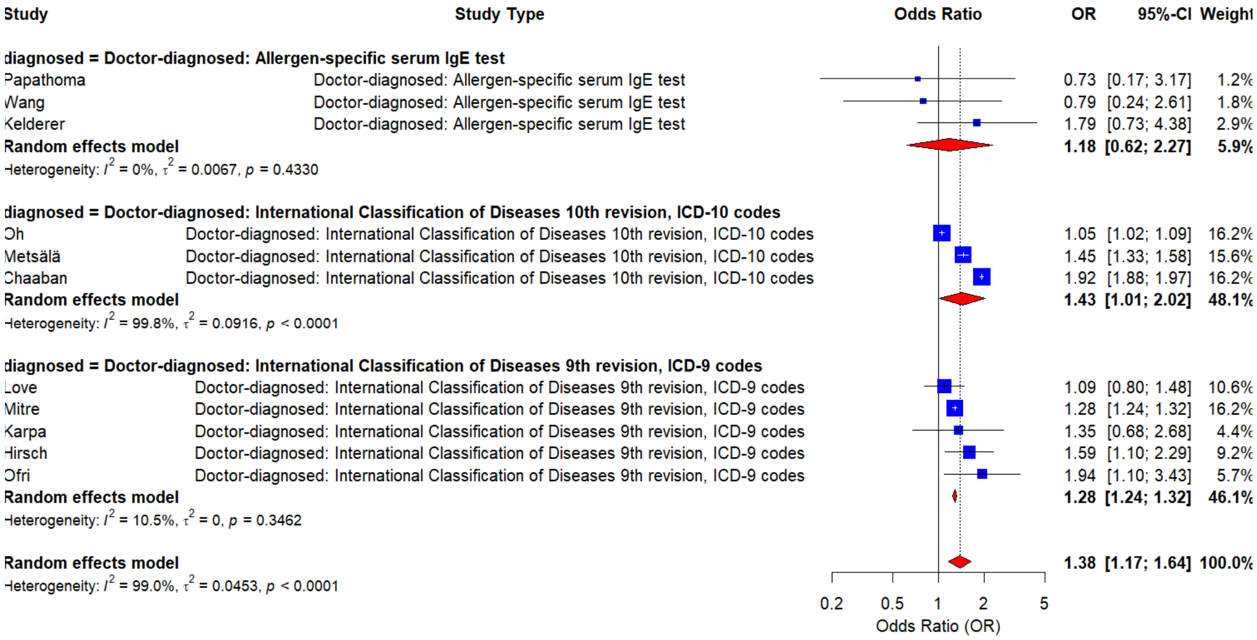

Supplementary Figure S19: Subgroup analysis of the association study type and food allergy according to acid-suppressive medications exposure.

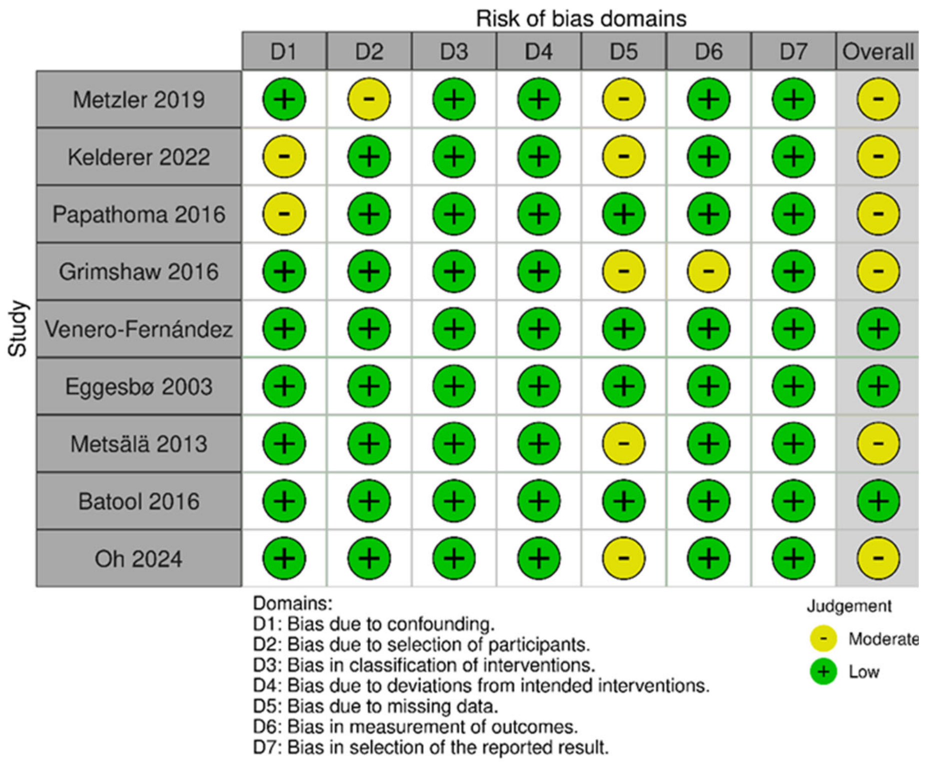

Supplementary Figure S20: Risk of bias of prenatal antbiotic exposure.

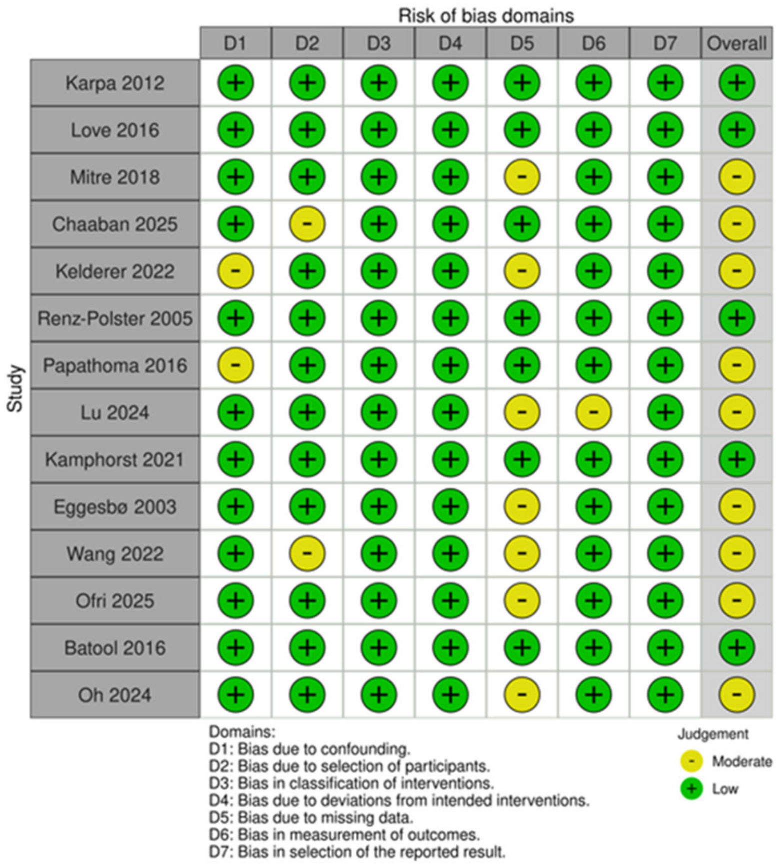

Supplementary Figure S21: Risk of bias of postnatal antbiotic exposure.

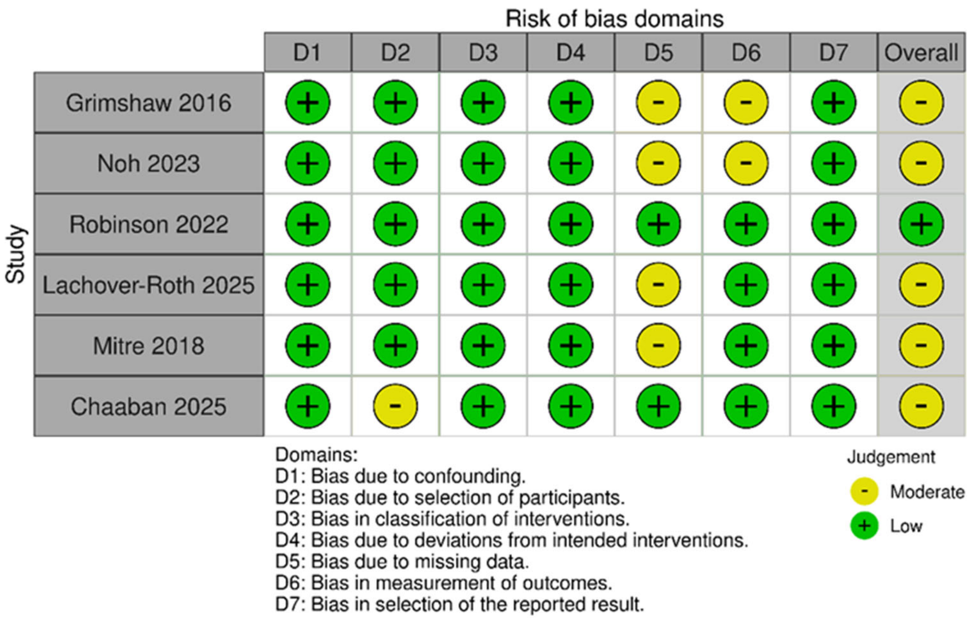

Supplementary Figure S22: Risk of bias of acid-suppressive medication exposure.

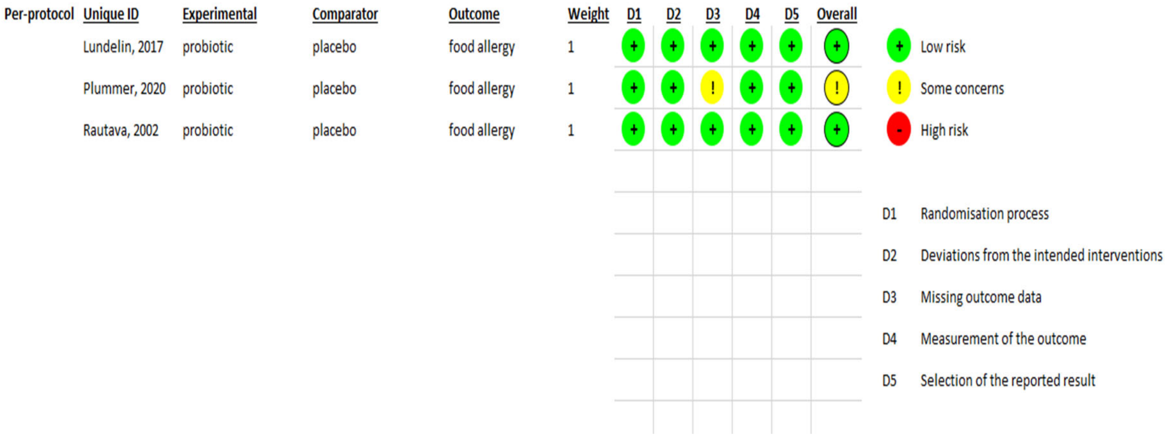

Supplementary Figure S23: Risk of bias of probiotic exposure.
